# A 45 X male patient with 7Q distal deletion and rearragement with SRY gene translocation; a case report

**DOI:** 10.1186/1687-9856-2013-S1-P190

**Published:** 2013-10-03

**Authors:** Ayşenur Ökten, Mevlit İkbal, Yakup Aslan

**Affiliations:** 1 KTU, Medical School Department of Pediatrics, Karadeniz Technical University, Turkey; 2 KTU, Medical School Department of Genetics, Karadeniz Technical University, Turkey

## 

We report a 3 month old boy, is the second child born to nonconsanguineous caucasian parents. He were delivered by cesarean section at 38 weeks gestation because of acute deterioration of heart rate, from 27 years old mother. He was hospitalised for respiratory distres and multiple congenital abnormalities. His birth weight was 2350 g (3-10 centile), lenght was 42 cm (below 3rd centile), and head circumference was 32 cm ( < 10 centile). Clinical examination showed respiratory distres, hypertension (85/41 mmHg), microcephaly, cleft lip and palate, low-set ears with large earlobes, anal stenosis and accessory nipple. The external genitalia was completaly normal male with a 2,5 cm lenght penil size and bilaterally descendent testis. Ophtalmic examination showed retinal coloboma and optic disc hypoplasia.

For to evaluate hypertension that refractory to medical treatment, urinary tract ultrasonography and MAG-3 scintigraphy were performed. Renal agenesia in the right side and severe dilatation of renal collecting system and ureter in the left side and thickening of bladder were detected. The urethral stenosis was found and corrected by cyctoscophy. The antihypertensive therapy decreased and the follow up USG showed improvement in ureteral diameter. He had abdominal operation with a misdiagnosis of necrotizing enterocolitis because of feeding intolerance, bloody stools and abdominal distension with a edematous intestinal loops on abdominal grapy. The laparocophy was revealed midgut malrotation and cecum was placed in the normal anatomic location. The magnetic resonans was showed only inferior cerebral vermis hypoplasia without any other defect.

Karyotype was 45,X without evidence of mosaicism.SRY gene (testis determining factor) was positive with polymerase chain reaction. FISH analysis showed SRY/distal 7q translocation. Testicular biopsy was not performed.

Discussion: The XX testicular disorder of sex development is rare condition, with a frequency of 1/20000-25000 male newborns, and 90% of the patients were positive for SRY gene on the X chrosome. Only few 45 X male patients were published to date.

The chromosome 7 q deletion syndrome is rare but well known condition, which shows broad clinical spectrum. All the malformation in our patient had been published with this syndrome. This syndrome may ocur as a result of simple deletion, but different aotosomal translocations had been also described. To our knowledge this is the first case of SRY gene translocation on distal 7q choromosome.

